# Abstract of Researches on Magnetism and on Certain Allied Subjects; Including a Supposed New Imponderable

**Published:** 1847-02

**Authors:** 


### PART VI.—SELECTIONS.

1. *Abstract of Researches on Magnetism and on certain allied Subjects; including a supposed new imponderable.* By Baron Von Reichenbach. Translated and abridged from the German, by Wm. Gregory, M. D., F. R. S., Prof. of Chemistry, &c.

As our limits will not allow us to give the whole of an interesting abstract of this work, published in the Jan., (1847) number of the American Journal of Medical Sciences, we give the following extracts from it, which will show the nature of the pretensions set up, which all will see to be closely allied to those of Mesmerism :

Baron Von Reichenbach thinks he has demonstrated that magnets act on the human body, especially in certain conditions ; and further, that he has demonstrated the existence, *in magnets*, of two forces, one which attracts iron and affects the needle, and one which acts on the nervous system, and which he has found unmixed *in crystals*. This new power, which he is disposed to view as the true agent in animal magnetism, he says, "is transferable from one body to another and is conductible through matter. A body may be, for a time, charged with it, and this is the true explanation of the fact, now demonstrated by the author, that a glass of water, as stated by Mesmer and his followers, may be magnetised by contact with a magnet, although that term is improper.

"But perhaps the most striking characters of this new power are, that it assumes, like electricity and magnetism, a polar arrangement in bodies, and that bodies charged with it are luminous, especially at the poles. The light, it is true, is only visible to certain sensitive individuals ; but not only are such persons of tolerably frequent occurrence, but the author has gone far towards demonstrating, that, although invisible to ordinary eyes, actual light, nevertheless, does emanate from the poles of powerful magnets.

"The author finds the new power in many unsuspected quarters, in the sun's rays, the moon's rays, heat, electricity, friction, and, above all, chemical action ; and the numerous and beautiful applications which at once suggest themselves, give a tenfold interest to this part of his researches. The human frame, especially the hand, whether in virtue of the incessant chemical changes going on in the body, or independently, is a rich source of the new power."

The new influence which the author thinks he has detected in magnets, in crystals, in light, heat, electricity, and chemical action, is due, he confesses, to the existence of his "new imponderable."

"Magnets of 10 lb. supporting power," says the author, "when drawn along the body, downwards, without contact, produce certain sensations in a certain proportion of human beings. Occasionally in 20, 3 or 4 sensitive individuals are found; and in one case, out of 22 females, examined by the author, 18 were found sensitive. The sensation is rather unpleasant than agreeable, and is like an *aura*, in some cases warm, in others cool; or it may be a pricking, or a sensation of the creeping of insects on the skin; sometimes headache comes rapidly on. These effects occur when the patient does not see the magnet, nor know what is doing; they occur both in males and females, although more fequently in females; they are sometimes seen in strong healthy people, but oftener in those whose health, though good, is not so vigorous, and in what are called nervous persons. Children are often found to be sensitive. Persons affected with spasmodic diseases, those who suffer from epilepsy, catalepsy, chorea, paralysis, and hysteria, are particularly sensitive. Lunatics and somnambulists are uniformly sensitive."

Healthy sensitive persons observe nothing further than the sensations above noticed, and experience no inconvenience from the approach of magnets. But the diseased sensitive subjects experience different sensations, often disagreeable, and occasionally giving rise to fainting, to attacks of catalepsy, or to very violent spasms. In such cases there occurs an extraordinary acuteness of the senses; smell and taste, for example, become astonishingly delicate and acute. The patients hear and understand what is spoken three or four rooms off, and their vision is often so irritable, that, while, on the one hand, they cannot endure the sun's light or that of a fire, on the other, they are able, in very dark rooms, to distinguish not only the outlines but also the colour of objects, where healthy people cannot distinguish anything at all.

The author's first series of recorded experiments refer to the appearance of light, visible only to the sensitive, at the poles and sides of powerful magnets.

"Mlle. N. being in catalepsy, insensible and motionless, but free from spasms, a horse-shoe magnet of 20 lb power was brought near to her head, when the hand attached itself so to the magnet, that whichever way the magnet was moved, the hand followed as if it had been a bit of iron adhering to it. She remained insensible, but the attraction was so powerful that when the magnet was removed in the direction of the feet, further than the arm could reach, she, still insensible, raised herself in the bed, and with the hands followed the magnet as far as she possibly could, so that it looked as if she had been seized by the hand, and that member dragged towards the feet. This was daily seen by the author between six and eight, P. M., when her attacks came on, in the presence of eight or ten persons, medical and scientific men. At other periods of the day when she was quite conscious, the phenomena were the same. She described the sensation as an irresistible attraction, which she felt compelled against her will to obey. The sensation was agreeable, accompanied with a gentle cooling aura streaming or flowing down from the magnet to the hand, which felt as if tied and drawn with a thousand fine threads to the magnet. She was not acquainted with any similar sensation in ordinary life." Similar results were obtained in other cases; in one case (the one quoted) a certain amount of attraction was also observed in the foot, but far weaker. It was found that the hand of the cataleptic patient had no attraction for iron filings, did not in the least affect the needle, and excited no appreciable attraction on a magnet, which was counterpoised on the beam of a balance, and brought near to the hand, although it required some force to prevent the hand from rising to the magnet. That is to say, while the magnet attracted the hand, as it were vitally, the hand did not attract the magnet statically."

In connexion with the foregoing is an article that more clearly defines all this class of phenomena than anything that has yet made its appearance, from which we take the following:

*The Power of the Mind over the Body; an experimental Inquiry into the Nature and Cause of the Phenomena attributed by Baron Reichenbach and others to a "New Imponderable."* By James Braid, M.R.C.S.E. (Edin. Med. & Surg. Jour. Oct. 1846.)

The preceding article contains an account of the wonderful, supposed, new discoveries of Baron Reichenbach, and in the present will be found the results obtained by Mr. Braid, on repeating the experiments of the former. This paper cannot fail to be read with deep interest. So curious and important does it seem to us, that we have not hesitated to give it nearly *in extenso*, and to appropriate to it far more space than is accorded to articles in this department of the Journal. It is one of the most valuable contributions to mental physiology and pathology that has for a long time been presented.

"On the first announcement," says Dr. Braid, "of Dr. Gregory's abstract of Baron Reichenbach's 'Researches on Magnetism,' I lost no time in procuring a copy, which I perused with intense interest. I had not proceeded far, however, when my experience with hypnotic patients enabled me to perceive a source of fallacy, of which the Baron must either have been ignorant, or which he had entirely overlooked.

From whatever cause this oversight had arisen, I felt confident that however carefully and perseveringly he had prosecuted his experiments, and however well calculated they had been for determining mere physical facts, still no reliance could be placed upon the accuracy of conclusions drawn from premises assumed as true, where especial care had not been taken to guard against the source of fallacy to which I refer, viz: the important influence of the mental part of the process, which is in active operation with patients during such experiments. I therefore resolved to repeat his experiments, paying the strictest attention to this point; and, as I had anticipated, the results were quite opposed to the conclusions of Baron Reichenbach. It is with considerable diffidence that I ventured to publish an opinion opposed to such high authority; but I shall briefly state the grounds of my own belief, and leave it to others to repeat the experiments, and determine which opinion is nearer the truth. The observations which I have to submit may, moreover, prove suggestive to others, and enable them not only to avoid sources of fallacy with which I am familiar, but may also lead to the detection of many which may have escaped my own observation.

"The great aim of Baron Reichenbach's researches in this department of science has been to establish the existence of a new imponderable, and to determine its qualities and powers in reference to matter and other forces, vital and inanimate. It unfortunately happens, however, that the only test of this alleged new force (with one solitary exception, and that as I thought by no means a satisfactory one), is the human nerve; and not only so, but it is further admitted that its existence can only be demonstrated by certain impressions imparted to, or experienced by, a comparatively small number of highly sensitive and nervous subjects. But it is an undoubted fact that with many individuals, and especially of the highly nervous, and imaginative, and abstractive classes, a strong direction of consciousness to any part of the body, especially if attended with the expectation or belief of something being about to happen, is quite sufficient to change the physical action of the part, and to produce such impressions from this cause alone, as Baron Reichenbach attributes to his new force. Thus every variety of feeling may be excited from an internal or mental cause—such as heat or cold, pricking, creeping, tingling, spasmodic twitching of muscles, catalepsy, a feeling of attraction or repulsion, sights of every form or hue, odours, tastes, and sounds, in endless variety, and so on, according as accident or intention may have suggested. Moreover, the oftener such impressions have been excited, the more readily may they be reproduced, under similar circumstances, through the laws of association and habit. Such being the fact, it must consequently be obvious to every intelligent and unprejudiced person, that no implicit reliance can be placed on the human nerve, as a test of this new power in producing effects from external impressions or influences, when precisely the same phenomena may arise from an internal or mental influence, when no external agency whatever is in operation.

"In order to guard against this source of fallacy, therefore, I considered it would be the best mode to throw patients into the nervous sleep, (the term adopted by me in preference to mesmeric sleep, for reasons to be explained presently,) and then operate on such of them as I knew had no use of their eyes during the sleep (for some have), and to take accurate notice of the results when a magnet capable of lifting fourteen pounds was drawn over the hand and other parts of the body without contact, after the manner described, as performed by Baron Reichenbach in his experiments.

"I experimented accordingly, and the results were, that in no instance was the slightest effect manifested, unless when the magnet was brought so near as to enable the patient to feel the abstraction of heat (producing a sensation of cold), when a feeling of discomfort was manifested, with a disposition to move the hand, or head, or face, as the case might be, from the offending cause. This indication was precisely the same when the armature was attached, as when the magnet was open: and in both cases, if I suffered the magnet to touch the patient, instantly the part was hurriedly withdrawn, as I have always seen manifested during the primary stage of hypnotism, when the patients were touched with any cold object. Now, inasmuch as patients in this condition, generally, if not always, manifest their perceptions of external impressions by the most natural movements, unless the natural law has been subverted by some preconceived notion or suggested idea to the contrary: and as I have operated with similar results upon a considerable number of patients, we have thus satisfactory proof that there was no real attractive power, of a magnetic or other nature, tending to draw the patient, or any of his members, so as to cause an adhesion between his body and the magnet, or between the latter and iron, as Baron Reichenbach had alledged. I conclude, therefore, that the phenomena of the apparent attraction manifested in his cases were due entirely to a mental influence; and I shall presently prove that this is quite adequate to the production of such effects.

"A lady, thirty years of age, was requested to hold out her right hand over the arm of an easy chair, whilst she turned her head to the left, to prevent her from seeing what I was doing, and to watch and describe to me the feelings she experienced in the hand during my process, which was to be performed without contact. She very soon felt a pricking in the point of the third finger, which increased in intensity and at length extended up the arm. I then asked her how her thumb felt, and presently the same feeling was transferred to it; and when asked to attend to the middle of the forearm, in like manner the feeling was presently perceived there. All the time I had been doing nothing; the whole was the result of her own mind acting on her hand and arm. I now took the large magnet, and allowed her to watch me drawing it slowly over the hand, when the feeling was much as before, only that she felt the cold from the steel when brought very near to the skin. It was precisely the same when closed as when opened, and the same sensations occurred when the north pole alone approximated, or the south alone, or both together. She experienced no sense of attraction between her hand and the magnet from either pole, nor from both combined. I now requested the lady to keep a steady gaze upon the poles of the large horse-shoe magnet, and tell me if she saw anything, (the room was not darkened nor was the light strong,) but nothing was visible. I then told her to look steadily, and she would see flame or fire come out of the poles. In a little after this announcement, she started, and said, 'Now I see it, it is red; how strange my eyes feel;' and instantly she passed into the hypnotic state. This lady had been repeatedly hypnotized. I now took the opportunity of testing her as to the alleged power of the magnet to attract her hand when asleep, but, as in other cases, the results were quite contrary—the cold of the magnet (and of either pole alike) caused her to withdraw her hand the moment it touched her. I now requested her to tell me what she saw (she being still in the sleep.) She said she still saw the red light. I desired her to put her finger to the place where she saw it. This she declined to do, being afraid that it would burn her. I thereupon assured her that it would not burn her, upon which she pointed to the same place where the magnet was held before she went into the sleep, instead of to where it was now held, which was near to her face, but towards the opposite side of the chair. This lady does not see from under her partially closed eyelids when hypnotized, as some patients do; and the evidence her testimony affords in support of my opinion upon this subject, is very conclusive, as she is a lady of very superior mental attainments, and one whose testimony merits unlimited confidence.

"I beg to call particular attention to the fact, that in this latter case, as with the fifth of the vigilant cases narrated, the first experiments were tried without any magnet or other object being pointed at or drawn over them, and still the mental influence was quite sufficient to change the physical action and produce decided and characteristic effects, where there could be no influence from without, of the nature alleged by Baron Reichenbach and the mesmerists.

"I had long been familiar with the fact, that during a certain stage of hypnotism, patients may be made to give various manifestations, or declarations of their feelings and emotions, according to previously existing ideas, or suggestions imparted to them during the sleep; and, moreover, that such associations once formed, were liable to recur ever after, under a similar combination of circumstances. As occurs in ordinary dreaming, they seem generally at once to adopt the idea as a reality, without taking the trouble of reasoning on the subject as to the probability of such ideas being only imaginary; and their extreme mobility at a certain stage of the hypnotic state, renders them prompt with their corresponding physical response. In proof of this, and how readily those attentive to these facts may misapprehend what they see realized in such cases, I beg to submit the following interesting illustration. When in London lately, I had the pleasure of calling upon an eminent and excellent physician, who is in the habit of using mesmerism in his practice, in suitable cases, just as he uses any other remedy. He spoke of the extraordinary effects which he had experienced from the use of magnets applied during the mesmeric state, and kindly offered to illustrate the fact on a patient who had been asleep all the time I was in the room, and in that stage, during which I felt assured she could overhear every word of our conversation. He told me that when he put the magnet into her hands, it would produce catalepsy of the hands and arms, and such was the result. He wafted the hands and the catalepsy ceased. He said that a mere touch of the magnet on a limb would stiffen it, and such he proved to be the fact.

"I now told him, that I had got a little instrument in my pocket, which, although far less than his, I felt assured would prove quite as powerful, and I offered to prove this by operating on the same patient, whom I had never seen before, and who was in the mesmeric state when I entered the room. My instrument was about three inches long, the thickness of a quill, with a ring attached to the end of it. I told him that when put into her hands, he would find it catalepsize both hands and arms as his had done, and such was the result. Having reduced this by wafting, I took my instrument from her, and again returned it, in another position, and told him it would now have the very reverse effect—that she would be able to hold it, and that although I closed her hands on it, they would open, and that it would drop out of them, and such was the case,—to the great surprise of my worthy friend, who now desired to be informed what I had done to the instrument to invest it with this new and opposite power. This I declined doing for the present; but I promised to do so, when he had seen some further proofs of its remarkable powers. I now told him that a touch with it on either extremity would cause the extremity to rise and become cataleptic, and such was the result; that a second touch on the same point would reduce the rigidity, and cause it to fall, and such again was proved to be the fact. After a variety of other experiments, every one of which proved precisely as I had predicted, she was aroused. I now applied the ring of my instrument on the third finger of the right hand, from which it was suspended, and told the doctor, that when it was so suspended, it would send her to sleep. To this he replied, "it never will," but I again told him I felt confident that it would send her to sleep. We then were silent, and very speedily she was once more asleep. Having aroused her, I put the instrument on the second finger of her left hand, and told the doctor that it would be found she would not go to sleep, when it was placed there. He said he thought she would, and he sat steadily gazing at her, but I said firmly and confidently that she would not. After a considerable time the doctor asked her if she did not feel sleepy, to which she replied "not at all;" could you rise and walk? when she told him she could. I then requested her to look at the point of the fore-finger of the right hand, which I told the doctor would send her to sleep, and such was the result; and, after being aroused, I desired her to keep a steady gaze at the nail of the thumb of the left hand, which would send her to sleep in like manner, and such proved to be the fact.

"Having repaired to another room, I explained to the doctor the real nature and powers of my little and apparently magical instrument,—that it was nothing more than my portmanteau-key and ring, and that what had imparted to it such apparently varied powers was merely the predictions which the patient had overheard me make to him, acting upon her in the peculiar state of the nervous sleep, as irresistible impulses to be affected, according to the results she had heard me predict."

